# Clinical characteristics and risk of all-cause mortality in low education patients with chronic obstructive pulmonary disease in the Chinese population

**DOI:** 10.7189/jogh.13.04163

**Published:** 2023-12-01

**Authors:** Qing Song, Cong Liu, Wei Cheng, Ling Lin, Tao Li, Xueshan Li, Xiao Liu, Yuqin Zeng, Rong Yi, Xin Li, Yan Chen, Shan Cai, Ping Chen

**Affiliations:** 1 Department of Respiratory and Critical Care Medicine, the Second Xiangya Hospital, Central South University, Changsha, Hunan, China; 2 Research Unit of Respiratory Disease, Central South University, Changsha, Hunan, China; 3 Diagnosis and Treatment Center of Respiratory Disease, Central South University, Changsha, Hunan, China; 4 Department of Pulmonary and Critical Care Medicine, Zhuzhou Central Hospital, Zhuzhou, Hunan, China; 5 Division 4 of Occupational Diseases, Hunan Prevention and Treatment Institute for Occupational Diseases, Changsha, Hunan, China

## Abstract

**Background:**

Education levels play a critical role in the development of chronic obstructive pulmonary disease (COPD), which mainly affects the elderly, who generally have a low level of education in China. We aimed to investigate the association between education level and COPD clinical characteristics and outcomes, especially the effects of education level on the all-cause mortality of COPD in the Chinese population.

**Methods:**

We retrieved data collected between December 2016 and June 2020 in the RealDTC, an ongoing multicenter, real-world study on the status of diagnosis and treatment of COPD. The patients were classified into low- and high-education groups. We extracted data on demographics, pulmonary function, Global Initiative for Chronic Obstructive Lung Disease (GOLD) grades, modified Medical Research Council (mMRC) scores, COPD Assessment Test (CAT) scores, exacerbation history, therapy, and comorbidities, and on mortality during three years of follow-up.

**Results:**

We included 4098 patients with COPD, of whom 3258 (79.5%) were of low education. This group had higher ages, CAT scores, mMRC scores, and numbers of exacerbations, as well as a greater proportion of females, never smokers, biofuel exposure, and GOLD grade 3. Logistic regression showed that being aged ≥65 years, being female, having biofuel exposure, having CAT scores of 20-29, and having ≥2 exacerbations were independently associated with having low education (*P* < 0.05). Furthermore, low-education COPD patients had a higher cumulative mortality risk during three years of follow-up than their high-education counterparts (hazard ratio (HR) = 1.75; 95% confidence interval (CI) = 1.17-2.61, *P* = 0.006).

**Conclusions:**

Low-education COPD patients, who accounted for most of our sample, had a higher symptom burden, risk of exacerbation, and risk of all-cause mortality. Clinicians attending COPD patients should be more attentive of individuals with low education levels.

Chronic obstructive pulmonary disease (COPD) is the most common chronic respiratory disease, characterised by persistent airflow limitation. It has high morbidity and mortality and significantly burdens society [1], especially in China, where it has a high prevalence of 13.7% in patients >40 years old [2].

Education levels play an important role in the development of COPD. For example, a study conducted in China found that patients with <9 years of education had higher COPD prevalence, and that education level is a risk factor for COPD independent of smoking [3]. Others found that a higher education level was associated with an overall lower risk of COPD, a higher probability of diagnosis, and a higher likeliness of receiving medications [4,5]. Lutter et al. [6] showed that lower education level was associated with lower forced expiratory volume in one second (FEV1), lower forced vital capacity (FVC) and FEV1/FVC, and higher Saint George’s Respiratory Questionnaire scores for COPD patients. Studies have also found a significant difference in education levels between the Chinese and other populations, confirming that low-education individuals (including middle school or under) for more than 80% of COPD patients overall [2,7]. However, the clinical characteristics of low-education COPD patients within the Chinese population have not been explored.

Another study found that higher education (>11 years) was associated with reduced mortality over the 4.5 years of follow-up, yet found no difference between education level and mortality after excluding COPD patients with asthma [6]. Lewis et al. [8] found that mortality for COPD was significantly increased for patients with below higher education levels after adjusting for age, sex, race, and smoking. However, they did not consider symptoms and pulmonary function, which were significantly associated with mortality. Lange et al. [9] found that shorter school education (compared to university education) was associated with a higher risk of COPD exacerbation and all-cause mortality. The underlying mechanism could be that people with low education levels engage in unhealthier behaviors and poor nutrition, leading to a high risk for COPD. Also, people with low education levels might have disadvantages in accessing health care services, possibly leading to worse health outcomes [10,11]. Although China is a developing country with a generally low level of education among people aged >50 years [12], the risk of all-cause COPD mortality in this group has not been studied.

We thus aimed to investigate the association between education level and COPD clinical characteristics and outcomes, including all-cause mortality, in the Chinese population.

## METHODS

### Study participants

We used data from the RealDTC (ChiCTR-POC-17010431) study on the clinical characteristics, diagnosis, treatment, and prognosis of COPD patients in the Chinese population, which recruited its first patient on 1 December 2016. Thirteen hospitals in the Hunan and Guangxi provinces participated, led by the Department of Respiratory and Critical Care Medicine at the Second Xiangya Hospital of Central South University. One national expert coordinated each research center and was responsible for local data collection and overall organisational management. The patients were diagnosed with COPD based on the Global Initiative for Chronic Obstructive Lung Disease (GOLD) 2017 report, a ratio of FEV1/FVC of <0.70 after inhaling a bronchodilator [13], and their data were recorded into a hospital database upon presentation. Data was collected between December 2016 and June 2020. We excluded patients with mental disorders who could not complete the questionnaire. We performed this study per the Declaration of Helsinki and received approval from the Ethics Committee of the Second Xiangya Hospital of Central South University (2016076). All patients provided their written informed consent.

### Data collection

We collected data on age, sex, education level, body mass index (BMI), smoke status, FEV1% predicted, FEV1/FVC, GOLD grades, GOLD groups, COPD Assessment Test (CAT) scores, modiﬁed Medical Research Council (mMRC) scores, number of exacerbations in the past year, inhalation therapy regimens and comorbidities (including asthma, bronchiectasis, lung cancer, chronic heart disease, hypertension, and diabetes) at the patient’s first hospital visit. We also recorded the data on mortality during three years of follow-up.

### Study procedure

We classified patients with COPD into low- and high-education groups. We defined the former as patients who received middle school or lower education (including middle school, primary school or lower, and education level <9 years), and the latter as patients who received at least high school education (including high school, college or higher, and education level ≥9 years) [2,3,5].

### Definition of variables

We defined exacerbation as a COPD progression that requires antibiotics, oral corticosteroids, or hospitalisation [14]. A current smoker was an individual who had smoking exposure of ≥10 packs/y, while a former smoker had ≥10 packs/y, but has not smoked for more than six months. We defined biofuel exposure as using biomass fuels (wood, grass, charcoal, or crop residues) for cooking or heating for at least two hours per day for at least one year [15]. According to the GOLD 2023 report, we assigned patients to three categories:

− Group A: 0-1 exacerbation per year, no hospitalisation, CAT scores of <10 and mMRC scores of 0 -1;
− Group B: 0-1 exacerbation per year, no hospitalisation, CAT scores of ≥10 or mMRC scores of ≥2;
− Group E: ≥2 exacerbations or ≥1 hospitalisation per year.

We based GOLD grades on the post-bronchodilator FEV1% [16]:

− GOLD 1: FEV1 ≥80% predicted;
− GOLD 2: FEV1 50-79% predicted;
− GOLD 3: FEV1 30-49% predicted;
− GOLD 4: FEV1 <30% predicted.

Prescription outcomes were continued use and adjustment treatment, with the latter defined as a change in the inhalation therapy drugs or stopping inhalation therapy drugs for more than three months during one year of follow-up [17].

### Statistical analysis

We used PASS software, version 15.0.5 (NCSS, LLC, Kaysville, Utah, USA) to calculate the sample size, with a proportion of low-education patients with COPD of 0.815 based on our previous study [7], a confidence level of 0.95, and a confidence interval (CI) width (two-sided) of 0.05. Considering a dropout rate of 20%, we estimated the minimum sample size at 1207.

We used the Kolmogorov-Smirnov test to verify the normality of the data, after which we compared normally distributed continuous variables with homogeneity of variance with Student’s *t-*test, and applied non-parametric tests otherwise. We analysed categorical variables with the χ^2^ test. We used logistic regression to analyse the relative factors for patients with low education and variables (*P-*value <0.05) and presented our findings as odds ratio (OR) and 95% CIs. We performed a multivariate Cox regression analysis to identify factors predicting mortality, using education level, age, sex, biofuel exposure, smoke status, BMI, CAT, mMRC, exacerbation in the past year, pulmonary function, therapy, prescription outcomes, and comorbidities as variables of interest. We also set up a survival curve between low-education and high-education groups during three years of follow-up. We considered *P* < 0.05 as statistically significant. We used SPSS 26.0 (IBM, Armonk, NY, USA) and Free Statistics software version 1.7.1 (Beijing, China) for all statistical analyses.

## RESULTS

### Clinical characteristics of low-education patients with COPD

**Figure 1:**
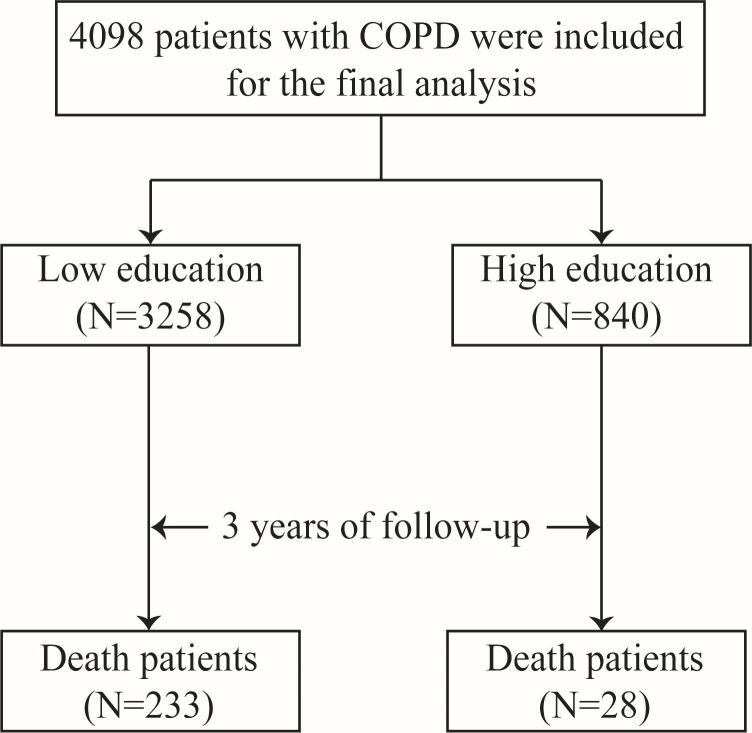
Flowchart. COPD, Chronic Obstructive Pulmonary Disease.

We included 4098 patients with COPD (**Figure 1**) with a mean age of 66.1 years (standard deviation (SD) = 9.1), of whom 86.3% were male and 3258 (79.5%) had a low education level.

Low-education patients with COPD had higher age, CAT scores, mMRC scores and number of exacerbations in the past year, and a greater proportion of females, never-smokers, as well as individuals with higher biofuel exposure, GOLD grade 3, GOLD group E, aged ≥65 years, CAT scores of 20-29, mMRC scores, and ≥2 exacerbations. However, they had a lower proportion of individuals with comorbidities, including asthma and chronic heart disease (*P* < 0.05) (**Table 1**).

**Table 1:** Clinical characteristics of low-education patients with chronic obstructive pulmonary disease

Variables	Total (n = 4098)	High education (n = 840)	Low education (n = 3258)	*P*-value
Age in years, mean (SD)	66.1 (9.1)	64.7 (9.6)	66.4 (8.9)	<0.05
Age, n (%)				<0.05
*<65*	1614 (39.4)	407 (48.5)	1207 (37.0)	
*≥65*	2484 (60.6)	433 (51.5)	2051 (63.0)	
Sex, n (%)				<0.05
*Male*	3537 (86.3)	768 (91.4)	2769 (85.0)	
*Female*	561 (13.7)	72 (8.6)	489 (15.0)	
BMI kg/m^2^, mean (SD)	22.5 (3.4)	22.7 (3.3)	22.5 (3.4)	0.158
BMI, n (%)				0.171
*<18.5*	483 (11.8)	89 (10.6)	394 (12.1)	
*18.5-23.9*	2286 (55.8)	458 (54.5)	1828 (56.1)	
*≥24*	1329 (32.4)	293 (349)	1036 (31.8)	
Smoke status, n (%)				<0.05
*Never-smoker*	990 (24.2)	159 (18.9)	831 (25.5)	
*Former-smoker*	1376 (33.6)	270 (32.1)	1106 (33.9)	
*Current-smoker*	1732 (42.2)	411 (49.0)	1321 (40.5)	
Smoking, pack/y, median (IQR)	30 (10-50)	36 (20-50)	30 (7.5-50)	<0.05
Biofuel exposure, n (%)				<0.05
*Yes*	1439 (35.1)	148 (17.6)	1291 (39.6)	
*No*	2659 (64.9)	692 (82.4)	1967 (60.4)	
FEV1% predicted, mean (SD)	52.7 (19.5)	53.6 (18.9)	52.5 (19.6)	0.172
FEV1/FVC, mean (SD)	47.7 (11.9)	48.1 (12.1)	47.6 (11.9)	0.347
GOLD grades, n (%)				<0.05
*1*	369 (9.0)	75 (8.9)	294 (9.0)	
*2*	1767 (43.1)	404 (48.1)	1363 (41.8)	
*3*	1475 (36.0)	264 (31.4)	1211 (37.2)	
*4*	487 (11.9)	97 (11.5)	390 (12.0)	
GOLD groups, n (%)				<0.05
*A*	492 (12.0)	137 (16.3)	355 (10.9)	
*B*	1619 (39.5)	339 (40.4)	1280 (39.3)	
*E*	1987 (48.5)	364 (43.3)	1623 (49.8)	
CAT, mean (SD)	15.6 (6.5)	14.2 (6.4)	15.9 (6.5)	<0.05
CAT, n (%)				<0.05
*<10*	777 (19.0)	208 (24.8)	569 (17.5)	
*10-19*	2252 (55.0)	475 (56.5)	1777 (54.5)	
*20-29*	963 (23.4)	141 (16.8)	822 (25.2)	
*≥30*	106 (2.6)	16 (1.9)	90 (2.8)	
mMRC, median (IQR)	2 (1-3)	2 (1-2)	2 (1-3)	<0.05
mMRC, n (%)				<0.05
*0-1*	1265 (30.9)	319 (38.0)	946 (29.0)	
*≥2*	2833 (69.1)	521 (62.0)	2312 (71.0)	
Therapy, n (%)				0.081
*LAMA*	1268 (30.9)	252 (30.0)	1016 (31.2)	
*LABA+LAMA*	58 (1.5)	14 (1.7)	44 (1.4)	
*LABA+ICS*	533 (13.0)	123 (14.6)	410 (12.6)	
*LABA+LAMA+ICS*	1967 (48.0)	382 (45.5)	1585 (48.6)	
Others*	272 (6.6)	69 (8.2)	203 (6.2)	
Exacerbation in the past year, median (IQR)	1 (0-2)	1 (0-2)	1 (0-2)	<0.05
Exacerbation in the past year, n (%)				<0.05
*0*	1704 (41.6)	397 (47.3)	1307 (40.1)	
*1*	956 (23.3)	205 (24.4)	751 (23.1)	
*≥2*	1438 (35.1)	238 (28.3)	1200 (36.8)	
Comorbidities, n (%)				
*Chronic heart disease*	44 (1.1)	15 (1.8)	29 (0.9)	<0.05
*Hypertension*	53 (1.3)	16 (1.9)	37 (1.1)	0.079
*Lung cancer*	34 (0.8)	7 (0.8)	27 (0.8)	0.990
*Diabetes*	24 (0.6)	7 (0.8)	17 (0.5)	0.309
*Bronchiectasis*	139 (3.4)	34 (4.0)	105 (3.2)	0.239
*Asthma*	994 (24.3)	243 (28.9)	751 (23.1)	<0.05

BMI – body mass index, COPD – chronic obstructive pulmonary disease, CAT – COPD assessment test, FEV1 – forced expiratory volume in one second, FVC – forced vital capacity, GOLD – Global Initiative for Chronic Obstructive Lung Disease, ICS – inhaled corticosteroids, IQR – interquartile range, LAMA – long-acting muscarinic antagonist, LABA – long-acting β2-agonist, mMRC – modiﬁed Medical Research Council, SD – standard deviation, SABA – short-acting β2-agonist, SAMA – short-acting muscarinic agonist

*Others including SAMA, SABA, SAMA+SABA, LAMA+ICS, ICS and no inhalation therapy.

### Factors correlated with the low education patients with COPD

We found that CAT scores of 20-29 (OR = 1.54; 95% CI = 1.17-2.01) and ≥2 exacerbations (OR = 1.23; 95% CI = 1.02-1.49) were positively associated with low education (*P* < 0.05), while comorbidities, including asthma (OR = 0.83; 95% CI = 0.69-0.99) and chronic heart disease (OR = 0.43; 95% CI = 0.22-0.84) were negatively associated (*P* < 0.05) (**Table 2**).

**Table 2:** Multivariate analysis of independently relative factors for low-education patients with chronic obstructive pulmonary disease*

Variables	OR (95% CI)	*P*-value
Age		
*<65*	ref	
*≥65*	1.34 (1.14-1.59)	<0.05
Sex		
*Male*	ref	
*Female*	1.43 (1.02-1.99)	<0.05
Biofuel exposure	1.67 (1.12-2.48)	<0.05
*No*	ref	
*Yes*	2.66 (2.19-3.24)	<0.05
CAT		
*<10*	ref	
*10-19*	1.14 (0.93-1.40)	0.219
*20-29*	1.54 (1.17-2.01)	<0.05
*≥30*	1.24 (0.69-2.25)	0.473
Exacerbation in the past year		
*0*	ref	
*1*	1.04 (0.86-1.27)	0.681
*≥2*	1.23 (1.02-1.49)	<0.05
Asthma	0.83 (0.69-0.99)	<0.05
Chronic heart disease	0.43 (0.22-0.84)	<0.05

ref- reference, CAT – COPD assessment test, OR – odds ratio, CI – confidence interval

*Adjusted for age, sex, biofuel exposure, CAT, exacerbation in the past year, smoke status, mMRC, GOLD grades, and comorbidities.

### Risk of all-cause mortality in low-education patients with COPD

**Figure 2:**
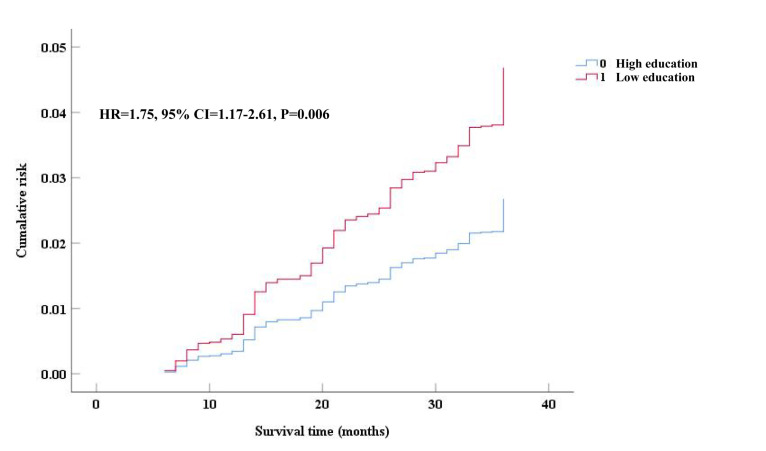
Cumulative risk of all-cause mortality between low education and high education patients with COPD during three years of follow-up. COPD – chronic obstructive pulmonary disease, CI – confidence interval, HR – hazard ratio.

A total of 261 (6.4%) patients with COPD died during three years of follow-up, of whom 233 (7.2%) had low education. Comparison of cumulative risk using the survival curve showed that low-education patients with COPD had a higher cumulative mortality risk compared with their high-education counterparts (hazard ratio (HR) = 1.75; 95% CI = 1.17-2.61, *P* = 0.006) (**Figure 2**).

### Multivariate analysis of risk factors for three years of all-cause mortality in patients with COPD

The Cox regression analysis demonstrated that the low education (HR = 1.75; 95% CI = 1.17-2.61), CAT scores of 20-29 (HR = 2.37; 95% CI = 1.40-4.01), CAT scores of ≥30 (HR = 4.43; 95% CI = 2.28-8.62), and mMRC scores of ≥2 (HR = 2.00; 95% CI = 1.33-3.00) were positively associated with three years of mortality, while a BMI≥24 kg/m^2^ (HR = 0.53; 95% CI = 0.36-0.79) and being female (HR = 0.60; 95% CI = 0.39-0.94) were negatively associated (*P* < 0.05) (**Table 3**).

**Table 3:** Cox regression analysis of relative factors for three years of all-cause mortality in patients with chronic obstructive pulmonary disease

Variables	HR (95% CI)	*P*-value
Education level		
*High education*	ref	
*Low education*	1.75 (1.17-2.61)	<0.05
Age		
*<65*	ref	
*≥65*	2.98 (2.10-4.23)	<0.05
Sex		
*Male*	ref	
*Female*	0.60 (0.39-0.94)	<0.05
Biofuel exposure		
*No*	ref	
*Yes*	1.35 (1.05-1.73)	<0.05
BMI		
*<18.5*	ref	
*18.5-23.9*	0.78 (0.57-1.08)	0.130
*≥24*	0.53 (0.36-0.79)	<0.05
CAT		
*<10*	ref	
*10-19*	1.55 (0.94-2.55)	0.089
*20-29*	2.37 (1.40-4.01)	<0.05
*≥30*	4.43 (2.28-8.62)	<0.05
mMRC		
*0-1*	ref	
*≥2*	2.00 (1.33-3.00)	<0.05
Lung cancer	6.83 (3.77-12.38)	<0.05
Diabetes	2.78 (1.01-7.66)	<0.05

ref – reference, BMI – body mass index, CAT – COPD assessment test, mMRC – modiﬁed Medical Research Council, HR – hazard ratio, CI – confidence interval

*Adjusted for education level, age, sex, biofuel exposure, smoke status, BMI, CAT, mMRC, exacerbation in the past year, pulmonary function, therapy, prescription outcomes, and comorbidities.

## DISCUSSION

In our study, low-education patients with COPD (middle school or lower) accounted for 79.5% of the total sample. This is in line with previous studies, where this population comprised approximately 80% of COPD patients [2,7,12,18], and is to be expected in the Chinese context, as most of the elderly have low education.

As the development of COPD was found to be independently associated with low education [19], it is necessary to determine the clinical characteristics of low-education patients for the prevention and treatment of COPD.

Similar to a previous study [9], low-education patients in our sample were older, had higher mMRC scores, and were mostly in the GOLD group E, but conversely comprised a lower proportion of current and a higher proportion of never smokers. Meanwhile, Miravitlles et al. [20] found that COPD patients of lower socioeconomic status had a higher prevalence of never smoking, similar to our results. This might be because low-education patients are financially unable to obtain more cigarettes, and because there were more female patients with low education in our sample, although there are relatively few female smokers in the Chinese population [2,12]. Simultaneously, this suggests that smoking cessation education should be strengthened for high-education patients with COPD, even though they might be more aware of the dangers of smoking.

Furthermore, low-education COPD patients in our study had a lower number of comorbidities, including asthma and chronic heart disease. Lutter et al. [6] also found that COPD patients with basic education (≤9 years) had a lower proportion of asthma than those with higher education (>11 years). This follows the findings of a prior study, which found that patients with higher education levels, higher annual household incomes, and good access to medical services had a higher probability of disease diagnosis [4]. However, our study is the first to identify several independently relative factors for low education patients with COPD, including age, biofuel exposure, sex, CAT scores, and exacerbation in the past year, which could help guide the prevention and treatment of COPD. Unlike Lutter et al. [6], we found that higher CAT scores were associated with low education in patients with COPD, possibly due to our inclusion of current smokers.

Although mortality is the most important factor in evaluating outcomes for COPD patients [21], the relationship between education levels and risk of all-cause mortality in this group was not fully explored in the Chinese population. We found that low-education COPD patients had a higher risk of all-cause mortality after controlling the confounding factors, including age, sex, biofuel exposure, smoke status, BMI, CAT, mMRC, exacerbation in the past year, pulmonary function, therapy, prescription outcomes, and comorbidities. Unlike Lutter et al. [6], they found no significant difference between education level and mortality after excluding COPD patients with asthma, possibly because low-education COPD patients accounted for most of our sample and had a higher risk of all-cause mortality. Eisner et al. [22] found that lower education and lower income were independently related to poor outcomes in this group, with a higher risk of acute COPD exacerbation. In a stratified analysis among current smokers, another study found that the mortality risk of screened COPD was high in those with lower education, higher income, living alone, and less social participation [23]. As low education level is an independent risk factor for COPD, clinicians should be more attentive to this population when educating them about disease management, especially as they might have a worse understanding of the disease and face challenges in obtainin better medical services, leading to poor outcomes [4,24].

This study has some limitations. We did not explore symptom changes, future risk of exacerbation, and hospitalisation among different education levels, which we will address in future research. We also did not include other indicators of socioeconomic status, such as income, occupation, and location of residence or housing, as we focused on the relationship between education level and COPD. Finally, we did not account for non-pharmacological treatment, including pulmonary rehabilitation, vaccination, and oxygen therapy; however, we previously found that the proportion of COPD patients treated non-pharmacologically was relatively small [7], so it is unlikely that this affected our findings.

## CONCLUSIONS

Lower-education individuals, who comprised most of the COPD patients in our sample, had a higher symptom burden and risk of exacerbation and a higher risk of all-cause mortality, suggesting clinicians should be more attentive to this population when treating COPD.
